# Variants of Monteggia Type Injury: Case Reports

**DOI:** 10.5704/MOJ.1503.001

**Published:** 2015-03

**Authors:** NAF Kamudin, M Firdouse, CS Han, A M Yusof

**Affiliations:** Faculty of Medicine, Universiti Sultan Zainal Abidin, Kuala Terengganu, Malaysia; * Orthopaedic Department, Hospital Sultan Haji Ahmad Shah, Temerloh, Malaysia

**Keywords:** Monteggia fracture-dislocation, hybrid lesion clinical examination and radiograph, surgery

## Abstract

**Background:**

Monteggia fracture-dislocation is rare in children. Various reports attest to its rarity, while recording the many variant of this injury. It is, therefore, easy to miss the diagnosis in the absence of proper clinical examination and radiographs.

**Case Report:**

This report highlights two rare variants of Monteggia fracture-dislocation seen in children. The first case was a 12-year old girl alleged to have fallen from a 15- feet tall tree and sustaining a combined type III Monteggia injury with ipsilateral Type II Salter-Harris injury of distal end radius with a metaphyseal fracture of the distal third of the ulna. The second case was a 13-year old who had sustained a closed fracture of atypical Type I Monteggia hybrid lesion, in a road traffic accident.

**Conclusion:**

This report highlights the rare variants of Monteggia fracture dislocation which could have been missed without proper clinical examinations and radiographs.

## Introduction

Monteggia fracture was originally described by Giovanni Batista Monteggia as an anterior dislocation of the head of radius and fracture of the proximal ulna^1^. The complexity of the mechanism of this injury has been described by various authors but Bado’s classification remains the most commonly used^1^. Various reports have shown that Monteggia fracture-dislocation is rare in children^2,3,4^

This is a report of two rare variants of Monteggia fracturedislocation. The first case is a combined type III Monteggia injury with ipsilateral Type II Salter-Harris injury of the distal end radius fracture with metaphyseal fracture of the distal third of the ulna and the second case is an atypical Type I Monteggia hybrid lesion.

## Case Reports

### First Case

A 12 years old girl, right hand dominant, allegedly fell from a tree of about 15 feet height landing on her left outstretched and hyper-pronated hand. She had no other injuries except for her left wrist and left elbow which were swollen, painful, with limited range of active motion. Clinical examination revealed swelling and deformity of her left wrist and left elbow, without neurovascular compromise. The initial radiograph showed Salter-Harris injury of distal end left radius, distal third left ulna metaphyseal fracture with ipsilateral proximal ulna fracture and left radial head posterior dislocation. The injured limb was rested in a backslab and planned for surgical intervention.

Under general anaesthesia. Intraoperative, patient was appropriately positioned and her left upper limb placed on arm board. Closed manipulative reduction was done under image intensifier guidance. The distal end of the left radius was first held with Kirschner wire. The distal left ulna fracture was aligned and was treated non-operatively. Then open reduction of the proximal left ulna using posterior approach was done and the fracture held with Kirschner wire. The left radial head was relocated using closed manipulative reduction. The limb was then immobilised in an above elbow resting plaster in 90 degree flexion and the forearm in full supination.

Patient was observed in the ward for compartment syndrome and subsequently discharged well. She was reviewed after three weeks post-operatively and repeated radiograph showed fracture callus formation. The K-wires were removed and she was started on range of motion exercises. At two months follow up patient had regained full flexion and extension of the elbow and wrist, with full pronation of the forearm, but limited forearm supination (0–60°). Radiographs showed all fractures had united.

**Fig. 1:**
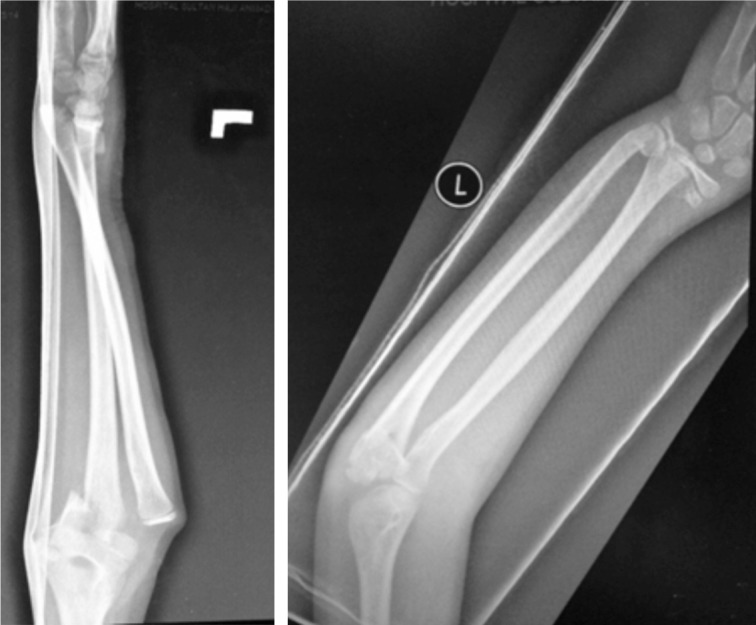
The initial radiograph taken in Emergency Department showing distal end radius Salter-Harris type II fracture with distal third metaphyseal ulna fracture with ipsilateral proximal ulna fracture with radial head posterior dislocation.

**Fig. 2:**
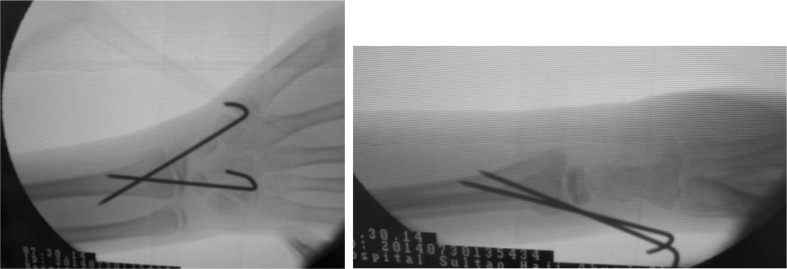
Radiograph under image intensifier guidance for distal end radius after reduction and K-wire insertion.

**Fig. 3:**
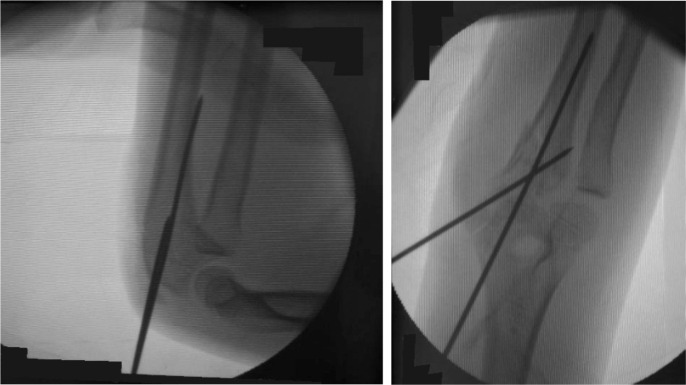
Radiograph of left elbow under image intensifier post--reduction and K-wire insertion.

**Fig. 4:**
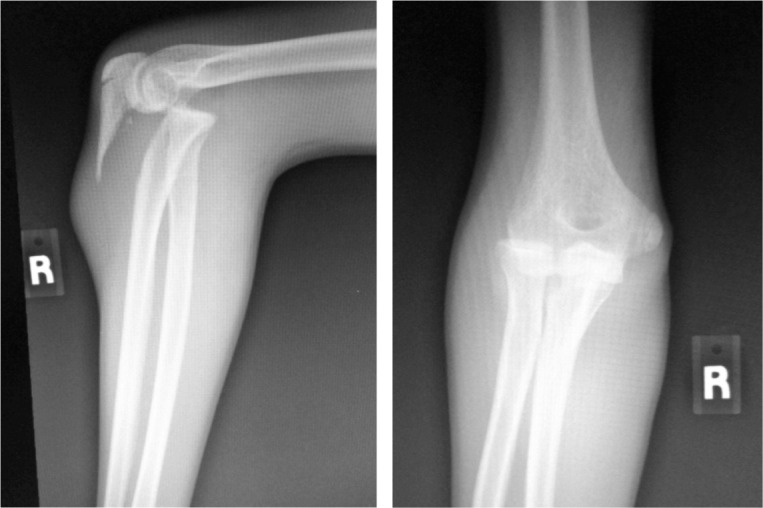
Initial radiograph taken in emergency department showing proximal ulna fracture with radial head anterior dislocation. Fracture of proximal ulna extending to olecranon articular surface.

**Fig. 5:**
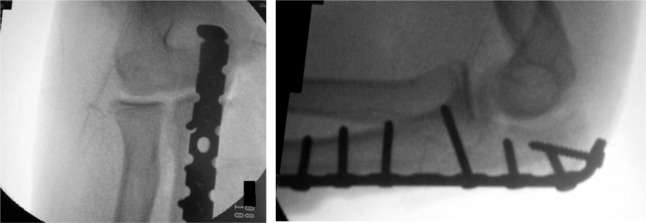
Radiograph post-plating proximal ulna under image intensifier showing good reduction.

### Second Case

A 13 years old boy, a right hand dominant, allegedly was involved in a road traffic accident and had fallen on his right outstretched hand. He had no other injuries except for pain and swelling of his right elbow. On examination the right elbow was deformed, tender on palpation, no compartment tightness -and good circulation. The initial radiographs showed proximal right ulna fracture with the fracture line extending into the elbow joint with right radial head anterior dislocation. The right upper limb was rested in a full length plaster slab and planned for surgery.

Under general anaesthesia, and right arm tourniquet, the patient was appropriately positioned and his right upper limb on an arm board. Procedure was done under general anaesthesia. Open reduction was done using the posterior approach for proximal right ulna. Reduction was done under image intensifier guidance and restoration of articular surface of olecranon was achieved. The right radial head was relocated by manipulation, and the reduction held with an 8- hole reconstruction plate and a lag screw was slotted through the plate to achieve compression over the fracture site, and reductions checked under image intensification

The patient was observed in the ward for 48 hours for compartment syndrome and the wound was inspected on the second postoperative day. Patient was discharged well and reviewed at 4 weeks, for wound inspection and rediograph. Patient was referred to physiotherapy for range of motion exercise of elbow, forearm and wrist. He was reviewed at 6 weeks, 3 months and 6 months post-operatively. He regained full functions at 6 months post operation, follow-up radiographs showed well united fractures.

## Discussion

Monteggia fracture–dislocation involving an ulna fracture in association with a radial head dislocation was classically described by Giovanni Monteggia based on the pattern of injuries in adults from cadaveric study^1^. Due to the infrequent occurrence of this type of injury it can be easily missed in paediatric patients if not specifically looked for^1^.

Numerous classification schemes have been subsequently described, but Bado’s classification has stood the test of time^1,2^. True or classical Bado’s classification divided Monteggia fracture-dislocations into four variants based on the direction of radial head dislocation. Several variants of the Monteggia injury have been further described in children and the commonest variant is Type 1 which is an anterior dislocation of the radial head (59%) and Type III lateral dislocation of radial head (26%).^2^

Bado further classified certain injuries as equivalent to the classic or true Monteggia lesion- in view of their similar mechanism of injuries, radiographic pattern, or methods of treatment These injuries were also known as Monteggia Equivalents Injuries Type I, II, III and IV^1^.

There was another description of the Monteggia injury which was a Monteggia lesion with anterior dislocation of the radial head, associated with a fracture of metaphyseal region of the ulna that extended into the olecranon, involving the intraarticular surface known as “Hybrid Lesion of Monteggia”^1^. Some cases of dislocation of the radial head in multiple directions were also reported^1^. The treatment and outcome of each fracture are determined by the direction of the radial head dislocation combined with the pattern of the ulnar fracture^1^.

It has been estimated that up to 50% senior house officers in accident and emergency departments and 25% of senior radiologists would miss a Monteggia injury^2^. In our two cases, the first case was referred to Orthopaedic Department by an A&E House Officer only as closed fracture of the distal end left radius. The second case was referred as closed fracture of the proximal right ulna alone. Both patients had painful forearms and elbow coupled with restricted elbow and forearm movements, which heightened the suspicion of Monteggia injuries. A good clinical examination of the elbow and forearm was therefore important to rule out this pattern of injury. Although in the acute setting, thorough clinical examination may be difficult in an uncooperative child it should nevertheless be routinely practised so as not to miss the variants of the Monteggia injury. Base on a high index of suspicion, appropriate full length radiographs should be further requested to identifying the injury and preventing its late complications.

In terms of treatment, nonoperative method has been reported with successful outcome^2,3^. Operative intervention is chosen if closed manipulative reduction failed or in unstable fracture dislocations^5^. Various reports have indicated that plastic deformity or incomplete Monteggia fracture-dislocation tend to be stable, thus treatment in plaster cast is sufficient to maintain acceptable anatomical position, achieving good results^2^

Regarding our first case, based on our interpretation of the literature, most cases reported were with incomplete fracture of proximal ulna^2^. In the present case however, the patient was diagnosed with combined Type III Montegia injury with ipsilateral Type II distal end left radius fracture and distal third left ulna metaphyseal fracture. The proximal left ulna was a comminuted fracture, thus open reduction was chosen to achieve anatomical reduction. The radial head was relocated using close reduction manipulation and found to be stable thus thus decided not for transcapitellar wiring to prevent further damage to capitellar and physeal area. The distal 3rd left ulna was aligned therefore decided for non operative treatment. Post operatively, the upper limb was maintained with left elbow in flexion and left forearm in full supination with resting plaster.

In the second case, patient was diagnosed with atypical Monteggia Type I Hybrid Lesion. The proximal right ulna fracture extended to the olecranon involving the articular surface, associated with anterior dislocation of the radial head. Open reduction and plating of proximal ulna was chosen to get anatomical reduction and to restore the articular surface of olecranon. The radial head was also relocated following the reduction of proximal ulna and was stable thus transcapitellar Kirschner wire was not inserted.

## Conclusion

Combined type III Monteggia injury with ipsilateral Type II Salter Harris of distal end radius with distal 3rd ulna metaphyseal fracture and Atypical Type I Monteggia Hybrid Lesion are two rare variants of Monteggia injury that are rarely reported. Both are unstable fractures and require surgical stabilization. Even though rare, this injury should not be missed. Thorough physical examination and a high index of suspicion especially in paediatrics patients are mandatory.
